# Evaluation of pediatric drugs on the surface roughness of primary enamel and restorative materials: an in vitro study

**DOI:** 10.1186/s12903-026-09009-8

**Published:** 2026-06-30

**Authors:** Manar Khalifa, Salwa M. Awad, Mostafa A. Abdelshafi

**Affiliations:** 1 Pediatric Dentistry Department, Faculty of Dentistry, Mansoura University, Mansoura, Egypt; 2 Dental Biomaterials Department, Faculty of Dentistry, Mansoura University, Mansoura, Egypt; 3 Prosthodontics Department, Dental Biomaterials Division, Faculty of Oral and Dental Medicine, Alsalam University, Tanta, Egypt

**Keywords:** Amoxicillin, Azithromycin, Surface roughness, Cention N, RMGI

## Abstract

**Background:**

Pediatric liquid medications may adversely affect the surface properties of dental tissues and restorative materials. This study assessed the effect of amoxicillin and azithromycin suspensions on the surface roughness of primary enamel, Cention N, and resin-modified glass ionomer (RMGI).

**Methods:**

A total of 90 specimens were included in this study: 30 primary enamel specimens, 30 Cention N specimens, and 30 RMGI specimens. Each substrate group was further subdivided into three subgroups (*n* = 10) according to the immersion medium: amoxicillin suspension, azithromycin suspension, or artificial saliva. Surface roughness (Ra) was measured using a contact profilometer at baseline and after completion of three consecutive 7-day immersion cycles separated by 2-day intervals. The cumulative exposure times were 126 min for amoxicillin and 42 min for azithromycin. Data were analyzed using IBM SPSS software. Normality was assessed using the Shapiro–Wilk test, and statistical analysis was performed using paired t-test, one-way ANOVA, and two-way ANOVA, with the level of significance set at *p* ≤ 0.05.

**Results:**

Both amoxicillin and azithromycin suspensions significantly increased surface roughness compared with artificial saliva (*p* < 0.001 and *p* = 0.001, respectively), with no significant difference between the two antibiotics (*p* = 0.999). In all material groups, both suspensions significantly increased surface roughness, whereas artificial saliva caused no significant change. Primary enamel showed a significantly lower increase in surface roughness than both Cention N and RMGI.

**Conclusions:**

Both amoxicillin and azithromycin pediatric suspensions increased the surface roughness of primary enamel, Cention N, and RMGI under the conditions of this in vitro study. Although azithromycin showed an alkaline pH, the comparable roughening effects of the two antibiotics suggest that surface changes may be influenced by multiple factors, including formulation characteristics and cumulative exposure time.

## Background

Pediatric liquid medications are widely prescribed for the management of infections, pain, and other common childhood conditions. Despite their therapeutic value, repeated exposure to these formulations may adversely affect dental hard tissues and restorative materials because of their pH, viscosity, sugar content, titratable acidity, and other formulation-related factors. Such effects are clinically important, as surface alterations may increase plaque retention, promote discoloration, and compromise the longevity of restorations [1–3].

Among the antibiotics frequently prescribed in pediatric practice, amoxicillin suspension (AMS) remains one of the most commonly used agents because of its effectiveness against a wide range of oral and systemic infections [4, 5]. Azithromycin suspension (AZS) may also be used in selected pediatric oral conditions and can be prescribed when an alternative to penicillin-based therapy is required [6]. It is available as a pediatric oral suspension and has the practical advantage of once-daily administration, whereas amoxicillin is commonly administered three times daily [7–9]. Therefore, comparing these two antibiotic suspensions is clinically relevant because they differ in formulation characteristics, pH, and dosing frequency.

Surface roughness is a clinically relevant property as it affects not only the esthetic appearance of dental surfaces but also their susceptibility to bacterial adhesion and biofilm accumulation. Increased roughness has been associated with greater plaque retention, gingival inflammation, discoloration, and a higher risk of caries development [1]. Therefore, evaluating surface roughness after repeated exposure to pediatric medications provides useful information about early surface deterioration and its possible clinical consequences. Primary enamel may be particularly susceptible to repeated chemical challenge, while restorative materials commonly used in pediatric dentistry may likewise undergo surface degradation after repeated exposure to liquid medications [10, 11].

In contemporary pediatric dentistry, adhesive restorative materials are commonly used to restore primary teeth because of their esthetics, conservative handling, and ion-releasing potential [12, 13]. Among these materials, Cention N has attracted attention as an alkasite restorative material with favorable mechanical properties and the ability to release fluoride, calcium, and hydroxyl ions under acidic challenge [14]. Resin-modified glass ionomer (RMGI) is also widely used in pediatric patients because it combines fluoride release and chemical adhesion with improved handling and resin reinforcement [15, 16]. Since both materials are frequently used in clinical pediatric practice, evaluating their behavior under exposure to pediatric liquid medications is of direct clinical relevance.

Although previous studies have evaluated the erosive or roughening effects of pediatric liquid medications, limited evidence is available regarding their effect on both primary enamel and contemporary restorative materials used in pediatric dentistry. In particular, data on the response of Cention N and RMGI to repeated exposure to pediatric antibiotic suspensions remain insufficient. Direct comparison between amoxicillin and azithromycin suspensions is also limited, especially when their different clinical dosing frequencies are considered.

Therefore, this in vitro study aimed to evaluate the effect of amoxicillin and azithromycin pediatric suspensions on the surface roughness of primary enamel, Cention N, and resin-modified glass ionomer using an immersion protocol based on the clinical dosing frequency of each antibiotic. The null hypothesis was that the change in surface roughness would not differ significantly among the tested immersion media or among the tested substrates.

## Methods

### Study design

This in vitro study evaluated the effect of pediatric antibiotic suspensions on the surface roughness of primary enamel and restorative materials.

### Ethics approval and consent to participate

Ethical approval was obtained from the Ethical Committee of Scientific Research, Faculty of Dentistry, Mansoura University, Egypt (Approval No. A01012024 PP). Informed consent was obtained from the parents or legal guardians prior to the collection of the teeth. All samples were anonymized and handled in accordance with ethical guidelines.

### Sample size calculation

Sample size was calculated using G*Power 3.1, with a significance level of 0.05 and a study power of 80%. Based on an effect size of 0.49 derived from a previous study [2], a minimum total sample size of 90 specimens was determined. Accordingly, nine subgroups were included, with 10 specimens allocated to each subgroup.

### Preparation of primary enamel specimens

Thirty intact naturally shed or extracted primary teeth were collected from the Pediatric Dentistry Outpatient Clinic, Faculty of Dentistry, Mansoura University. Teeth with caries, developmental defects, previous restorations, visible enamel cracks, craze lines, or structural defects were excluded. Teeth were examined using a magnifying loupe at 5× magnification before specimen preparation. The collected teeth were disinfected in 0.1% thymol solution for 24 h, cleaned with pumice paste and a prophylaxis brush, and stored in artificial saliva until use. Each tooth was embedded in an acrylic resin mold (4 × 4 cm), with the labial surface left exposed. After embedding, the exposed labial enamel surfaces were finished and polished under water irrigation using TOR VM discs (TOR VM Ltd., Moscow, Russia) in a sequential manner from coarse to superfine abrasiveness. The specimens were then sequentially numbered and randomly assigned to the study groups using an online randomization program.

### Preparation of restorative material specimens

Table 1 summarizes the restorative materials and immersion media used in this study. For each restorative material, 30 disc-shaped specimens (4 mm in diameter and 2 mm in thickness) were fabricated using split Teflon molds. The materials were manipulated in accordance with the manufacturers’ instructions. Each material was inserted into the mold, covered with Mylar strips, and compressed between two glass slides to remove excess material and produce standardized flat surfaces.

**Table 1:** Tested restorative materials and immersion media used in the study

Materials	Type	Composition	Manufacturer	Batch/Lot No
Fuji II LC	Resin Modified Glass Ionomer (RMGI) Cement	**Powder**: 100% fluoroaluminosilicate **Liquid**: 35% HEMA, 25% distilled H_2_O, 24% polyacrylic acid, 6% tartaric acid besides 0.10% camphorquinone.	GC; Tokyo-Japan	2,401,251
Cention N	Alkasite Restorative Material	UDMA, Aromatic aliphatic-UDMA, DCP, PEG-400 DMA Barium aluminosilicate glass, Calcium barium aluminum fluorosilicate glass, Calciumfluoro silicate glass, Ytterbium trifluoride, Isofiller.	Ivoclar Vivadent AG, Schaan, Liechtenstein	Z03CKR
Artificial Saliva	Artificial Saliva	Methyl-phydroxybenzoate (2.0 g), KCl, Sodium carboxymethyl cellulose , MgCl₂0.6 H₂O, CaCl₂0.2 H₂O (0.166 g), K₂HPO₄ pH adjusted to 7.00 with KOH	Prepared at Faculty of pharmacy, Mansoura University-Egypt	-
E-MOX 250 mg/5 ml Suspension	Oral antibiotic suspension (amoxicillin)	Amoxicillin trihydrate Sucrose, carboxymethylcellulose, Caramel oil, Citric acid colloidal silicon dioxide, lactose monohydrate flavour oil and sodium benzoate	EIPICO (Egypt)	2,505,170
Zithrokan 200 mg/5 ml suspension	Oral antibiotic suspension azithromycin	Active: Azithromycin dihydrate Inactive: Sucrose, avicel mannitol, colloidal silicon dioxide, and flavourings	Hikma Pharmaceuticals (Egypt)	1582

Cention N was mixed according to the manufacturer’s instructions, inserted as a single increment, and light cured for 10 s using an LED curing unit (Elipar S10; 3 M ESPE Co., Seefeld, Germany) with an irradiance of 1200 mW/cm². Fuji II LC capsules were mixed for 10 s at 4000 rpm using an amalgamator, inserted into the mold, and light cured for 10 s using the same LED curing unit. After removal from the molds, all restorative specimens were finished and polished under water irrigation using the same TOR VM disc sequence, from coarse to superfine abrasiveness. No surface protective varnish or coating was applied to the Fuji II LC specimens to permit direct assessment of the effect of the tested immersion media on the material surface. The specimens were then stored in artificial saliva at 37 °C for 24 h before baseline surface roughness measurement. All restorative procedures were carried out by a single trained operator to ensure procedural standardization and reduce operator-dependent variability.

### Grouping of specimens

**Fig. 1:**
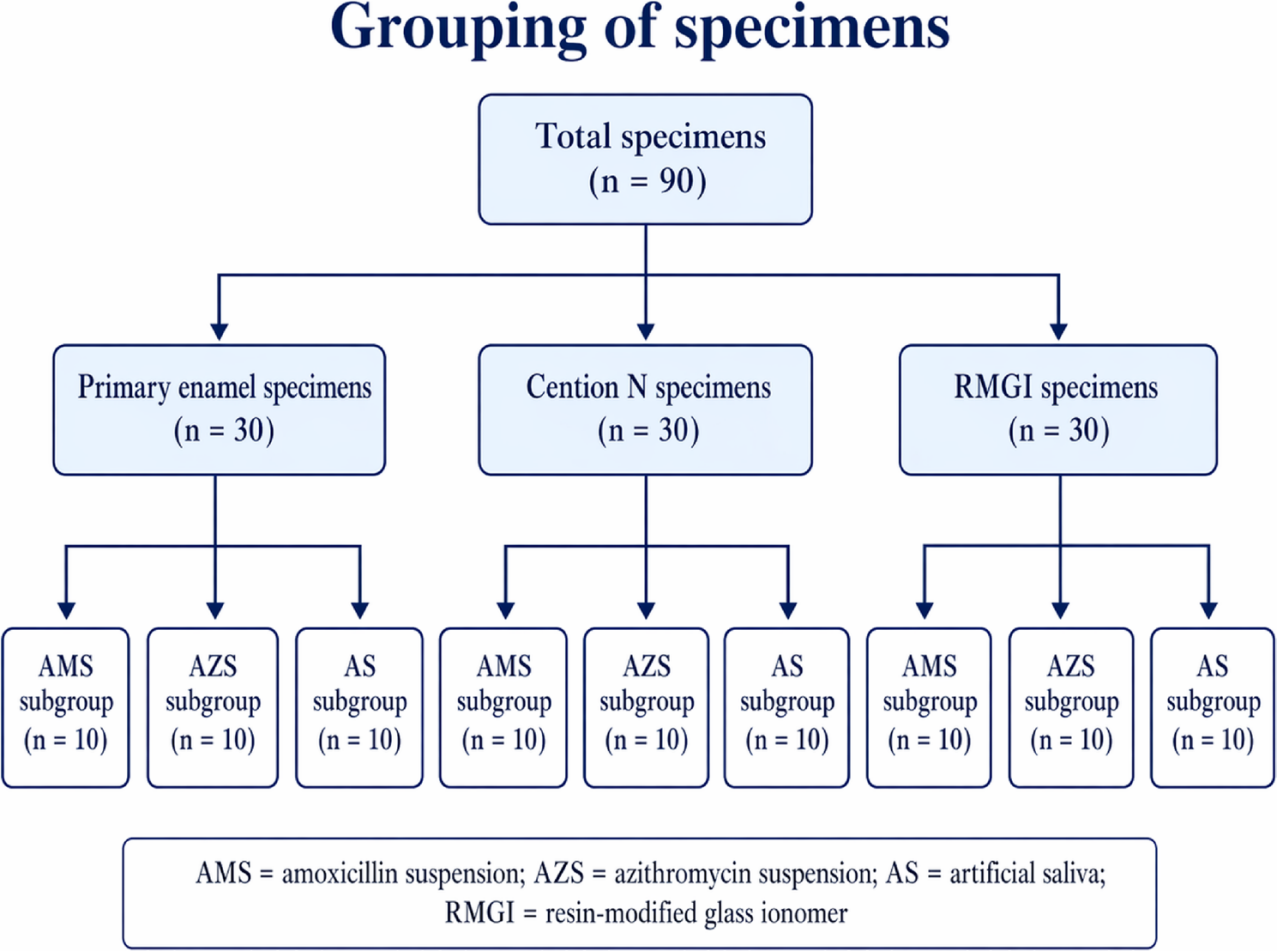
Flowchart illustrating specimen grouping and subgroup allocation

A total of 90 specimens were included in the study: 30 primary enamel specimens, 30 Cention N specimens, and 30 RMGI specimens. Each main group was further subdivided into three subgroups (*n* = 10) according to the immersion medium: amoxicillin suspension (AMS), azithromycin suspension (AZS), or artificial saliva (AS) (Fig. 1).

### Preparation of artificial saliva and pH measurement

Artificial saliva was prepared according to the formulation described by McKnight-Hanes and Whitford, with the exclusion of sorbitol to reduce viscosity [17]. The solution was freshly prepared at the Faculty of Pharmacy, Mansoura University. Its composition per liter was as follows: methyl-p-hydroxybenzoate, 2.00 g; potassium chloride, 0.625 g; sodium carboxymethyl cellulose, 10.00 g; magnesium chloride hexahydrate, 0.059 g; calcium chloride dihydrate, 0.166 g; and dipotassium hydrogen phosphate, 0.804 g. The pH was adjusted to 7.00 using potassium hydroxide.

Two commercial pediatric antibiotic suspensions were used: Zithrokan (200 mg/5 mL) as the azithromycin suspension and E-MOX (250 mg/5 mL) as the amoxicillin suspension. The pH of each immersion medium was measured at room temperature (25 °C) using a calibrated electrode pH meter (Beckman Instruments, Fullerton, CA, USA). Before measurement, the pH meter was calibrated according to the manufacturer’s instructions using standard buffer solutions of pH 4.01, 7.01, and 10.01. The antibiotic suspensions were reconstituted according to the manufacturers’ instructions. After complete reconstitution, each suspension was manually shaken for 30 s before pH measurement and before each subsequent reading to ensure homogeneity. The electrode was rinsed with distilled water and gently dried between readings to avoid cross-contamination. For each immersion medium, three consecutive readings were obtained, and the values were recorded as mean ± standard deviation. The measured pH values were 10.20 ± 0.07 for azithromycin suspension, 6.90 ± 0.10 for amoxicillin suspension, and 7.00 ± 0.08 for artificial saliva.

### Immersion protocol

After baseline surface roughness measurement, specimens were subjected to three consecutive 7-day immersion cycles separated by 2-day intervals, resulting in 21 exposure days. According to the clinical dosing frequency of each medication, specimens in the amoxicillin subgroup were immersed for 2 min, three times daily, while specimens in the azithromycin subgroup were immersed for 2 min, once daily. Thus, the total cumulative exposure time was 126 min for amoxicillin and 42 min for azithromycin.

After each immersion, specimens were rinsed with distilled water and stored in artificial saliva at 37 °C. The artificial saliva was renewed daily. Control specimens were stored in artificial saliva only, which was also renewed daily [2]. Surface roughness was reassessed after completion of the third immersion cycle.

### Surface roughness measurement

Surface roughness was measured using a contact profilometer (Mitutoyo SJ-210, Japan). For each specimen, three consecutive readings were taken in different directions, and the mean Ra value was calculated. Measurements were obtained at baseline and after completion of the immersion protocol. The profilometer was operated at a cut-off value of 0.25 mm, a stylus speed of 0.5 mm/s, and a sampling length of 2.5 mm. The device was calibrated before each measurement session to ensure measurement accuracy [18].

### Statistical analysis

Data were analyzed using IBM SPSS Statistics for Windows, version 27.0 (IBM Corp., Armonk, NY, USA). Normality was checked with the Shapiro–Wilk test. Quantitative data were presented as mean ± standard deviation, with minimum and maximum values where relevant. Statistical significance was set at *p* ≤ 0.05. Paired t-tests were used for within-subgroup comparisons between baseline and post-immersion surface roughness. The increase in surface roughness was then evaluated by two-way ANOVA to assess the effects of material type, immersion medium, and their interaction. Where variance homogeneity was not met according to Levene’s test, Welch’s ANOVA followed by Games-Howell post hoc test was used for pairwise comparisons.

## Results

**Fig. 2:**
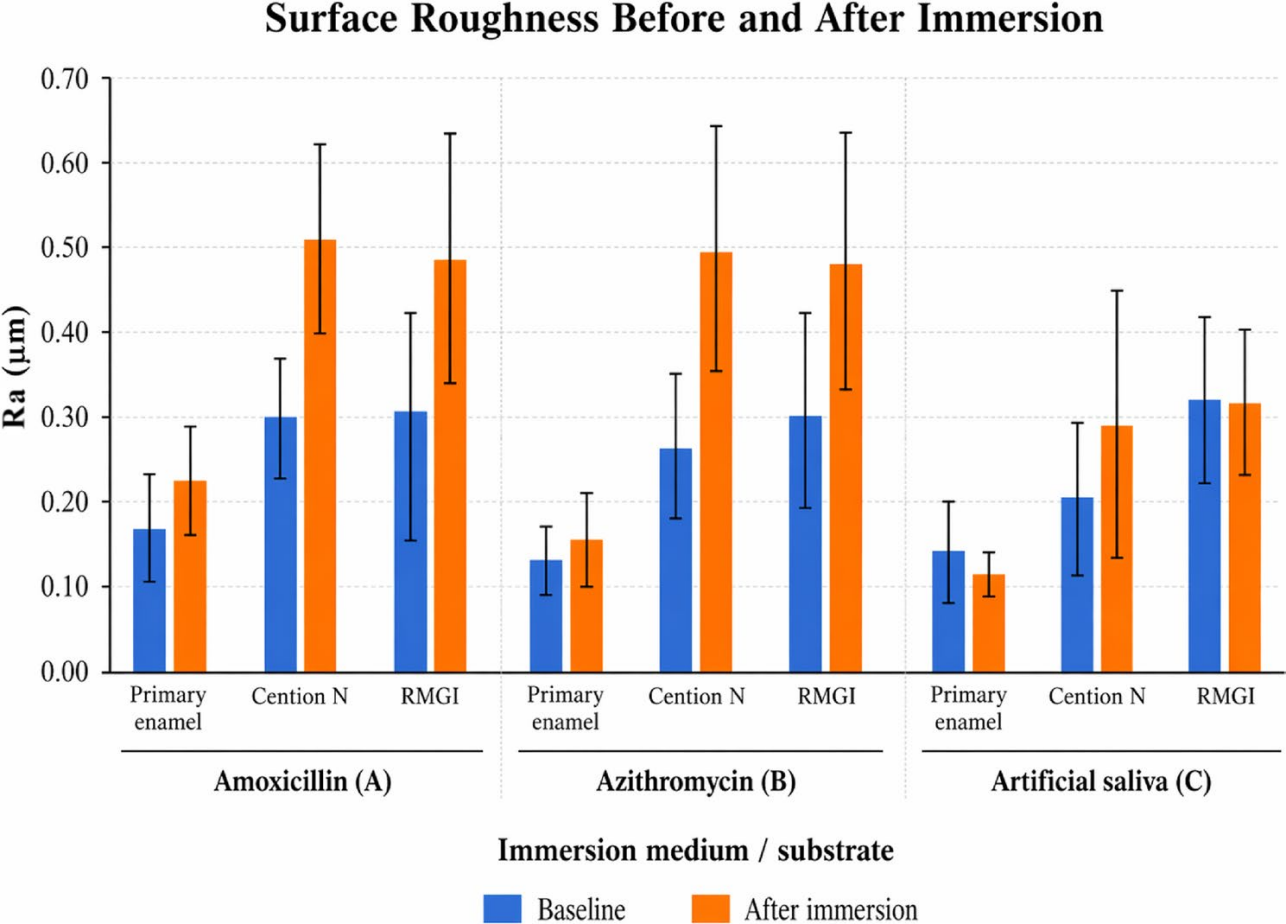
Mean surface roughness (Ra) values of primary enamel, Cention N, and RMGI at baseline and after immersion in amoxicillin suspension, azithromycin suspension, and artificial saliva. Bars represent mean values and error bars indicate standard deviation

Within the primary enamel group, both amoxicillin and azithromycin caused significant increases in surface roughness after immersion compared with baseline values (*p* = 0.003 and *p* = 0.019, respectively), whereas artificial saliva produced no significant change (*p* = 0.063). A similar pattern was observed for Cention N, in which significant increases were detected after exposure to amoxicillin and azithromycin (*p* = 0.001 for both), while the change after artificial saliva was not statistically significant (*p* = 0.081). For the RMGI group, both amoxicillin and azithromycin also resulted in significant increases in surface roughness (*p* < 0.001 and *p* = 0.008, respectively), whereas artificial saliva showed no significant effect (*p* = 0.977) (Table 2; Fig. 2).

**Table 2:** Intragroup comparison of surface roughness values before and after immersion in the tested media

Material	Immersion medium	Surface Roughness		t	*p*
		Baseline (Ra0), mean ± SD (µm)	After immersion (Ra1), mean ± SD (µm)		
Primary enamel	Amoxicillin	0.171 ± 0.058	0.231 ± 0.056	4.087^*^	0.003^*^
	Azithromycin	0.129 ± 0.040	0.158 ± 0.050	2.857^*^	0.019^*^
	Artificial saliva	0.142 ± 0.051	0.115 ± 0.018	2.117	0.063
Cention N	Amoxicillin	0.300 ± 0.065	0.505 ± 0.105	5.221^*^	0.001^*^
	Azithromycin	0.265 ± 0.080	0.497 ± 0.131	4.595^*^	0.001^*^
	Artificial saliva	0.206 ± 0.086	0.292 ± 0.154	1.962	0.081
RMGI	Amoxicillin	0.307 ± 0.108	0.488 ± 0.136	6.507^*^	< 0.001^*^
	Azithromycin	0.301 ± 0.109	0.483 ± 0.138	3.429^*^	0.008^*^
	Artificial saliva	0.324 ± 0.095	0.324 ± 0.075	0.029	0.977

Data are presented as mean ± SD (*n* = 10). Ra = average surface roughness

Paired t-test was used for intragroup comparisons

Statistically significant at *p* ≤ 0.05

Two-way ANOVA revealed statistically significant effects of both material type and immersion medium on the increase in surface roughness (*p* < 0.001 for both). In contrast, the interaction between material type and immersion medium was not statistically significant (*p* = 0.392), indicating that the effect of the tested immersion media followed a generally similar pattern across the studied substrates (Table 3).

**Table 3:** Two-way ANOVA for the increase in surface roughness

Source	Type III Sum of squares	df	Mean Square	F	*p*-value	Partial Eta Squared
Corrected Model	0.747	8	0.093	7.765	< 0.001^*^	0.434
Intercept	0.996	1	0.996	82.841	< 0.001^*^	0.506
Material Type	0.365	2	0.182	15.169	< 0.001*	0.272
Immersion Media	0.332	2	0.166	13.811	< 0.001^*^	0.254
Material type × immersion medium	0.050	4	0.013	1.040	0.392	0.049
Error	0.974	81	0.012			
Total	2.718	90				
Corrected Total	1.722	89				

Two-way ANOVA was used to evaluate the effects of material type and immersion medium, as well as their interaction, on the increase in surface roughness

R² = 0.434; adjusted R² = 0.378. Statistically significant at *p* ≤ 0.05

Post hoc comparison among the immersion media showed that both amoxicillin and azithromycin produced greater increases in surface roughness than artificial saliva. Pairwise comparison using Games-Howell post hoc test demonstrated that the increase in surface roughness was significantly higher in the amoxicillin group than in the artificial saliva group (*p* < 0.001), and significantly higher in the azithromycin group than in the artificial saliva group (*p* = 0.001). No statistically significant difference was found between amoxicillin and azithromycin (*p* = 0.999), indicating a comparable roughening effect of the two antibiotic suspensions. The mean increase in surface roughness was 0.149 ± 0.109 for amoxicillin, 0.148 ± 0.157 for azithromycin, and 0.019 ± 0.105 for artificial saliva (Table 4).

**Table 4:** Comparison of the increase in surface roughness among the tested immersion media

Variable	Amoxicillin (*n* = 30)	Azithromycin (*n* = 30)	Artificial saliva (*n* = 30)
Increase in surface roughness, min–max	-0.083 to 0.382	-0.036 to 0.552	-0.119 to 0.434
Increase in surface roughness, mean ± SD	0.149 ± 0.109	0.148 ± 0.157	0.019 ± 0.105
Pairwise comparisons (Games–Howell)	A vs. B: *p* = 0.999	A vs. C: *p* < 0.001*	B vs. C: *p* = 0.001*

Data are presented as mean ± SD

Pairwise comparisons were performed using Games–Howell post hoc test. A = amoxicillin; B = azithromycin; C = artificial saliva

*Statistically significant at *p* ≤ 0.05

Post hoc comparison among the tested substrates showed that primary enamel exhibited a significantly lower increase in surface roughness than both Cention N and RMGI (*p* < 0.001 and *p* = 0.003, respectively), whereas no statistically significant difference was found between Cention N and RMGI (*p* = 0.349) (Table 5).

**Table 5:** Comparison of the increase in surface roughness among the tested substrates

Variable	Primary enamel (*n* = 30)	Cention *N* (*n* = 30)	RMGI (*n* = 30)
Increase in surface roughness, min–max	-0.089–0.148	-0.086–0.552	-0.119–0.428
Increase in surface roughness, mean ± SD	0.021 ± 0.053	0.174 ± 0.151	0.121 ± 0.145
Pairwise comparisons	A vs. B: *p* < 0.001*	A vs. C: *p* = 0.003*	B vs. C: *p* = 0.349

Data are presented as mean ± SD

Pairwise comparisons were performed using the appropriate post hoc test. A = primary enamel; B = Cention N; C = resin-modified glass ionomer (RMGI)

*Statistically significant at *p* ≤ 0.05

## Discussion

The present study demonstrated that both amoxicillin and azithromycin pediatric suspensions significantly increased the surface roughness of primary enamel, Cention N, and RMGI compared with artificial saliva. Although the two suspensions differed in measured pH, no statistically significant difference was detected between them in terms of their roughening effect. In addition, two-way ANOVA showed that both immersion medium and material type significantly influenced the increase in surface roughness, whereas the interaction between them was not significant. Therefore, the null hypothesis was rejected, as significant differences were found among the tested immersion media and among the tested substrates. However, the non-significant interaction indicates that the effect of the tested media followed a broadly similar pattern across the studied substrates. Overall, primary enamel showed a lower increase in surface roughness than the restorative materials.

Surface roughness was selected as the primary outcome because it reflects changes in surface quality that may favor plaque retention, bacterial adhesion, and discoloration, and may ultimately affect the clinical performance of restorative materials [19–21]. For this reason, a reliable method for roughness assessment was required. In the present study, contact profilometry was used because it provides practical and reproducible quantitative measurements of surface change [22–24]. However, surface roughness alone does not provide microscopic or chemical information about the exact degradation mechanism; therefore, it should be considered a clinically relevant screening parameter for early surface deterioration after repeated chemical exposure [25].

In the present study, amoxicillin and azithromycin produced comparable increases in surface roughness despite their different measured pH values, suggesting that pH alone cannot fully explain the observed surface changes. The alkaline pH of azithromycin may have contributed to surface roughening, particularly in resin-containing materials such as Cention N and RMGI. In resin-based materials, alkaline exposure may promote water sorption and hydrolytic degradation of the resin matrix and silane coupling layer, weakening the filler-matrix interface and leading to filler exposure or partial filler loss. These changes may increase surface irregularity [26]. However, the similar roughening effect observed with amoxicillin indicates that other factors should also be considered. Previous studies have shown that pediatric liquid medications may affect dental surfaces even when their pH is not strongly acidic, suggesting that titratable acidity or alkalinity, viscosity, sugar content, soluble solids, excipients, and exposure frequency may also contribute to surface alteration [3, 27–30]. Therefore, pH should be interpreted as one contributing factor rather than the sole determinant of erosive or roughening potential.

The lack of a significant difference between amoxicillin and azithromycin may also be partly explained by the immersion regimen. In the present study, amoxicillin was applied three times daily, whereas azithromycin was applied once daily, reflecting their clinical dosing frequencies. Across the three 7-day cycles, the cumulative exposure time was 126 min for amoxicillin and 42 min for azithromycin. Therefore, the greater exposure frequency of amoxicillin may have offset, at least in part, the difference in pH between the two suspensions and contributed to their comparable roughening effects. This suggests that surface alteration is influenced not only by pH, but also by cumulative exposure time and formulation-related factors [2, 31].

The increase in surface roughness observed in primary enamel may be attributed to repeated superficial mineral alteration after cyclic exposure to the tested suspensions. Although primary enamel is highly mineralized, repeated contact with pediatric liquid medications may disrupt its surface integrity and create early topographic irregularities that become evident as increased roughness. This interpretation is consistent with previous reports describing erosive changes in primary enamel after exposure to pediatric liquid medicaments [27–31].

Likewise, the increase in surface roughness observed in RMGI agrees with Hasan et al., who reported deterioration in the surface quality of glass ionomer–based restorative materials after exposure to pediatric medications [2]. This behaviour is plausible because RMGI contains both an acid-base reactive glass ionomer phase and a resin component, making it susceptible to water-mediated surface alteration, matrix degradation, and filler exposure under repeated chemical challenge [15, 16]. The resin component of RMGI may undergo water sorption and hydrolytic degradation, while the glass ionomer phase may be affected by ion exchange and superficial dissolution [32]. These combined effects may explain the increased surface roughness observed after exposure to the antibiotic suspensions.

In contrast, comparable evidence for Cention N under pediatric medication challenge remains limited, which adds relevance to its inclusion in the present study. The significant roughness increase observed in this material suggests that newer alkasite-based restoratives are also vulnerable to repeated chemical exposure. Cention N contains a resin-based matrix combined with alkaline glass fillers capable of releasing ions, particularly under acidic conditions. Although this composition is designed to provide favorable mechanical properties and ion-releasing potential, repeated exposure to liquid medications may still affect the surface integrity of the material. Water sorption may lead to superficial softening or degradation of the resin matrix, weakening of the silane coupling layer, and exposure or partial dislodgement of filler particles. These changes may create surface irregularities and explain the increase in Ra values observed after repeated immersion. Therefore, the roughening of Cention N may be related to the combined effect of its resin matrix, filler system, and repeated chemical challenge [11, 14, 33]. This supports the significant main effect of material type observed in the present study and indicates that substrate composition influenced the magnitude of roughness change.

Post hoc comparison further showed that primary enamel exhibited a significantly lower increase in surface roughness than both Cention N and RMGI, whereas no significant difference was found between the two restorative materials. This suggests that, under the conditions of the present study, the restorative materials were more susceptible to surface alteration than primary enamel [34, 35]. This may be attributed to compositional differences between enamel and restorative materials. Primary enamel is predominantly mineralized, whereas Cention N and RMGI contain matrix phases that may undergo superficial degradation under repeated chemical challenge, leading to matrix loss, filler exposure, and a greater increase in surface roughness [34, 36, 37].

The clinical relevance of the observed Ra changes should also be considered. A surface roughness value of approximately 0.2 μm has commonly been used as a reference threshold above which bacterial plaque accumulation may increase [38]. In the present study, several post-immersion Ra values, especially for Cention N and RMGI, exceeded this reference value. This suggests that repeated exposure to pediatric antibiotic suspensions may increase the risk of plaque retention, staining, and surface deterioration of restorations. However, this threshold should be interpreted as a clinically relevant reference point rather than an absolute cutoff, as clinical plaque accumulation is also influenced by salivary flow, oral hygiene, pellicle formation, diet, and intraoral clearance [39].

The non-significant interaction between material type and immersion medium suggests a broadly similar response to the tested suspensions across the studied substrates. Nevertheless, the greater increase in surface roughness observed in the restorative materials indicates greater susceptibility to repeated medication exposure than primary enamel under the present conditions. This may be clinically relevant in children receiving repeated or prolonged liquid antibiotic therapy in the presence of existing restorations.

### Limitations

The findings should be interpreted within the limitations of an in vitro design, which cannot fully reproduce oral conditions such as salivary flow, pellicle formation, oral clearance, brushing, diet, and oral hygiene practices. Only two commercial pediatric antibiotic formulations were tested, and surface roughness was the only outcome evaluated; therefore, other properties such as microhardness, color stability, solubility, ion release, and microscopic surface morphology require further investigation. In addition, the different cumulative exposure times between amoxicillin and azithromycin reflected clinical dosing frequency but limited direct chemical comparison between the two suspensions.

## Conclusion

Within the limitations of this in vitro study, both amoxicillin and azithromycin pediatric suspensions increased the surface roughness of primary enamel, Cention N, and RMGI compared with artificial saliva. No significant difference was found between the two antibiotics; however, this finding should be interpreted cautiously because the suspensions differed in pH and cumulative exposure time. Repeated exposure to pediatric liquid antibiotics may therefore affect the surface quality of primary enamel and commonly used pediatric restorative materials.

## Data Availability

All data generated or analyzed during this study are included in this published article. Additional data are available from the corresponding author “Mostafa A. Abdelshafi” on reasonable request.

## Abbreviations

AMS: Amoxicillin suspension
ANOVA: Analysis of variance
AS: Artificial saliva
AZS: Azithromycin suspension
DCP: Dicumyl peroxide
HEMA: 2-Hydroxyethyl methacrylate
PEG-400 DMA: Polyethylene glycol 400 dimethacrylate
Ra: Average surface roughness
RMGI: Resin-modified glass ionomer
CN: Cention N
SD: Standard deviation
UDMA: Urethane dimethacrylate
